# Foraging Ecology of Fall-Migrating Shorebirds in the Illinois River Valley

**DOI:** 10.1371/journal.pone.0045121

**Published:** 2012-09-18

**Authors:** Randolph V. Smith, Joshua D. Stafford, Aaron P. Yetter, Michelle M. Horath, Christopher S. Hine, Jeffery P. Hoover

**Affiliations:** 1 F. C. Bellrose Waterfowl Research Center, Forbes Biological Station, Illinois Natural History Survey, Prairie Research Institute, University of Illinois at Urbana-Champaign, Havana, Illinois, United States of America; 2 Illinois Natural History Survey, Prairie Research Institute, University of Illinois at Urbana-Champaign, Champaign, Illinois, United States of America; Australian Wildlife Conservancy, Australia

## Abstract

Populations of many shorebird species appear to be declining in North America, and food resources at stopover habitats may limit migratory bird populations. We investigated body condition of, and foraging habitat and diet selection by 4 species of shorebirds in the central Illinois River valley during fall migrations 2007 and 2008 (Killdeer [*Charadrius vociferus*], Least Sandpiper [*Calidris minutilla*], Pectoral Sandpiper [*Calidris melanotos*], and Lesser Yellowlegs [*Tringa flavipes*]). All species except Killdeer were in good to excellent condition, based on size-corrected body mass and fat scores. Shorebird diets were dominated by invertebrate taxa from Orders Diptera and Coleoptera. Additionally, Isopoda, Hemiptera, Hirudinea, Nematoda, and Cyprinodontiformes contribution to diets varied by shorebird species and year. We evaluated diet and foraging habitat selection by comparing aggregate percent dry mass of food items in shorebird diets and core samples from foraging substrates. Invertebrate abundances at shorebird collection sites and random sites were generally similar, indicating that birds did not select foraging patches within wetlands based on invertebrate abundance. Conversely, we found considerable evidence for selection of some diet items within particular foraging sites, and consistent avoidance of Oligochaeta. We suspect the diet selectivity we observed was a function of overall invertebrate biomass (51.2±4.4 [SE] kg/ha; dry mass) at our study sites, which was greater than estimates reported in most other food selection studies. Diet selectivity in shorebirds may follow tenants of optimal foraging theory; that is, at low food abundances shorebirds forage opportunistically, with the likelihood of selectivity increasing as food availability increases. Nonetheless, relationships between the abundance, availability, and consumption of Oligochaetes for and by waterbirds should be the focus of future research, because estimates of foraging carrying capacity would need to be revised downward if Oligochaetes are truly avoided or unavailable for consumption.

## Introduction

Populations of many shorebird species appear to be declining throughout North America [1]–[5]. The mid-continent region of the United States is primarily used by shorebirds as stopover habitat during migration [2], [5]–[7]. Thus, the best way to affect shorebird fitness in this region is through management of habitat quantity and quality [2], [5]–[7]. Supporting this notion is evidence that migration habitat quality can influence shorebird populations [8], [9], and migratory patterns [10]. Because shorebirds may spend little time at individual stopover locations, their energy demands should require them to forage efficiently and opportunistically [11]–[13]. Indeed, several researchers have reported shorebirds using this foraging strategy [11]–[13]. Additionally, optimal foraging theory may predict that animals would forage opportunistically when food resources are abundant [14], [15], and we suspect food resources to be abundant at most highly used stopover locations [6], [16].

Wetlands in the mid-continent region are critically important to shorebirds as “refueling” habitats during migrations between Central American wintering areas and arctic breeding grounds [7], [17]. Previous investigations of shorebird foraging ecology have largely been conducted outside of the Upper Mississippi River and Great Lakes Region Joint Venture (hereafter JV) focal region [5], [12], [13], [18]–[22]. Few researchers have investigated foraging ecology of shorebirds in the mid-continent region [23], [24], or existing studies were of limited scale (i.e., [25], [26]). However, to emphasize the area’s importance, Chautauqua National Wildlife Refuge (hereafter CNWR) lies within the Illinois River valley (IRV) focus area [27] and has been designated a Western Hemisphere Shorebird Reserve Network site. CNWR may host 100,000–250,000 shorebirds annually during fall [28], and ≥5% of the global Pectoral Sandpiper (*Calidris melanotos*) population migrates through Illinois annually [29]. Understanding shorebird foraging ecology at this important bird area could help guide conservation planning throughout mid-latitude migration areas by identifying characteristics associated with use and selection (*see* [30∶20]).

The U.S. Shorebird Conservation Plan identified several research priorities to stimulate investigation of these long-distance migrants [2]. Many of these included some aspect of foraging ecology, including analyses of dietary requirements and preferences, and studies elucidating the relationship between wetland use and forage characteristics [2]. Similarly, Oring et al. [30] suggested investigation of resource use by highly congregated shorebirds was needed to improve our understanding of migratory stopover sites and the potential for foraging habitat to limit populations. Finally, the JV Shorebird Conservation Strategy identified food abundance, diet, and energetic carrying capacity for migrating shorebirds as specific research needs to improve shorebird conservation in this region [5].

We framed our research to address questions developed by the U.S. Shorebird Conservation Plan [2] and the JV [5], which were pertinent to our study area. Specific topics included, shorebird health in relation to habitat, diet composition of different shorebird foraging guilds, and food availability and abundance at migration areas [5∶36]. Therefore, we studied the foraging ecology of Pectoral and Least Sandpipers (*Calidris minutilla*), Lesser Yellowlegs (*Tringa flavipes*), and Killdeer (*Charadrius vociferus*) during fall migration. We collected foraging shorebirds and substrate core samples from foraging sites to estimate food abundance at shorebird-collection and random locations during fall migrations 2007 and 2008 within selected wetlands in the central IRV. Our objectives were to: 1) estimate body condition of migrating Least and Pectoral Sandpipers, Lesser Yellowlegs, and Killdeer during fall; 2) identify foods consumed by the 4 target species and evaluate their relative importance, and; 3) use data on invertebrate foods from shorebird diets and core samples to investigate potential selection of foraging patches within wetlands (i.e., third-order selection; [31]) and diet items at specific foraging sites (i.e., fourth-order selection). We predicted that: 1) shorebird mass (corrected for structural size) would be within published ranges, based on the high-quality habitat we perceived to exist in our study area; 2) shorebirds would select foraging patches within wetlands based on food abundance, as food availability can influence habitat use [10], [32], and; 3) that the diets of individual birds would consist of items relative to their availability in foraging locations.

## Methods

### Ethics Statement

We made every attempt to reduce disturbance, stress, and other impacts to target specimens and all other local fauna. We collected specimens using standard protocols and followed the Standard Conditions for Federal Migratory Bird Scientific Collecting Permit (United States Fish and Wildlife Service regulations 50 CFR 21.23). The University of Illinois at Urbana-Champaign Institutional Animal Care and Use Committee approved our experimental protocols (protocol number 06211). Collections were made under authorization of the United States Fish and Wildlife Service (scientific collection permit number MB145466-1) and the Illinois Department of Natural Resources (scientific permit numbers NH07.4071, NH08.4071, and NH09.4071). Permission and authorization to conduct research on, and remove habitat samples from, state-owned and managed property was issued by the Illinois Department of Natural Resources (research permit numbers SS07-49, and SS08-36). Authorization to work on CNWR was granted by the United States Fish and Wildlife Service (special use permit number 33653-07-06). Permits were not required to conduct research, collect specimens or habitat samples from privately owned property, but collections were included under the jurisdiction of the state and federally issued collection permits. Appropriate permission was received prior to entering or working on private lands.

### Study Area

Our study sites included backwater lakes and wetlands associated with the LaGrange Pool of the Illinois River (river miles 80.2–157.6) in Fulton, Mason, and Tazewell counties, Illinois. The importance of these floodplain wetlands to migratory waterbirds has been described in detail [33]–[35]. Many wetlands in our study area were managed to promote moist-soil vegetation, an important food for migratory waterfowl [36]. Moist-soil management typically requires natural or managed dewatering of wetlands to expose mud flats during the growing season. Thus, the region commonly provides abundant foraging habitat for shorebirds during fall migration.

**Figure 1:**
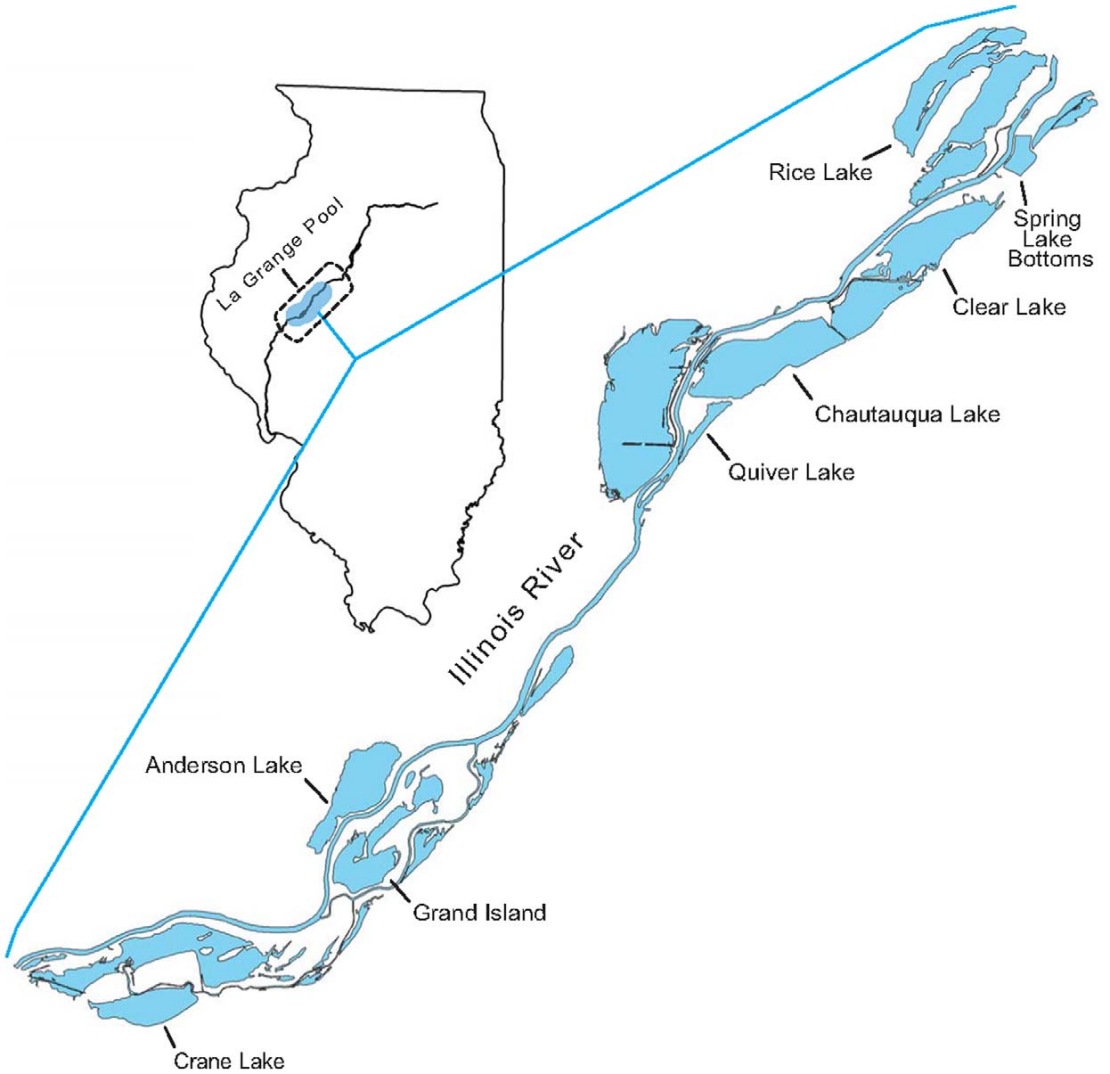
Map depicting our study area within La Grange Pool (dotted line) of the Illinois River in central Illinois, and specific study wetlands (labeled).

CNWR (Figure 1) was considered the most important of our collection sites and may host substantial numbers of shorebirds during migration [28]. Other publicly- and privately-owned and managed wetlands in the IRV also receive considerable use by fall-migrating shorebirds. Areas managed by the Illinois Department of Natural Resources (IDNR) included: Rice Lake, Anderson Lake, and Spring Lake State Fish and Wildlife Areas (Figure 1). Privately-owned wetlands included Grand Island, Crane Lake and Clear Lake (Figure 1). Finally, unmanaged backwater wetlands occasionally drawdown naturally and attract foraging shorebirds. Therefore, we collected shorebirds at 1 unmanaged wetland, Quiver Lake, at which water levels were dictated by the Illinois River (Figure 1). Typical habitat features of these sites included at least one large (200–925 ha) bottomland lake that was at least partially dewatered during summer (all sites except Quiver Lake). Five sites (Anderson, Clear, Crane, Rice, and Spring lakes) also had smaller (15–100 ha) leveed impoundments that were managed independently of the larger bottomland lakes. Water levels at all sites varied within and among years due to precipitation, fluctuating levels of the Illinois River, and site-specific management actions. Therefore, we were able to collect birds in only one of the two years at most sites, but did collect birds during both years at 2 sites (Grand Island, Clear Lake).

### Field

We collected foraging Killdeer, Least and Pectoral Sandpipers, and Lesser Yellowlegs with shotguns and non-toxic shot (Hevi-shot ®, Environ-metal, Inc.) during July and August 2007–2008 (Table 1). We observed feeding shorebirds for ≥10 minutes prior to collection to ensure they had not been feeding at another location and that they contained sufficient food for analysis. Immediately following collection, we injected a 10% buffered formalin solution into the upper digestive tract of each bird to stop digestion and placed a plastic cable-tie around the neck at the base of the head to prevent loss of ingesta. We uniquely labeled and bagged each bird and placed them in a cooler until we could transport them to the laboratory for processing (≤6 hours). We recorded the location of each collected bird using a handheld GPS unit and removed a wetland substrate core sample from the feeding location (5 cm diameter and 5 cm depth; [37]). Following all daily collections, we used a random numbers table to select an easting and northing distance (m) for each bird and collected a core sample from this randomly-selected location. Thus, each collected shorebird was paired with 2 core samples; one taken from the feeding site and one at a random location within the wetland (hereafter, “collection-site” and “random”, respectively). We preserved and stored all core samples in plastic bags with 10% buffered formalin solution stained with rose bengal until processed in the laboratory.

**Table 1:** Number of Killdeer (KILL), Least Sandpipers (LESA), Lesser Yellowlegs (LEYE), and Pectoral Sandpipers (PESA) collected and included in analyses of size-corrected body mass by site, year, and species.

	Species				
Study Site and Year	KILL	LESA	LEYE	PESA	Total
Chautauqua Lake 2007	12	16	24	29	81
Clear Lake 2007	5	0	3	6	14
Grand Island 2007	15	14	12	4	45
Quiver Lake 2007	3	6	0	0	9
Total 2007	35	36	39	39	149
Anderson Lake 2008	2	6	4	25	37
Clear Lake 2008	7	13	12	0	32
Crane Lake 2008	7	2	1	7	17
Grand Island 2008	0	0	15	4	19
Rice Lake 2008	6	5	0	0	11
Spring Lake 2008	12	3	0	0	15
Total 2008	34	29	32	36	131
Grand Total	69	65	71	75	280

### Laboratory Methods

We weighed shorebirds (±0.1 g) and recorded structural measurements to compute size-corrected body mass (hereafter, body mass) indices and scored body fat content using the Monitoring Avian Productivity and Survivorship (MAPS) method [38]. We placed hemostats at the proximal end of the esophagus and distal end of the proventriculus to prevent mixing or loss of ingesta prior to removal. Gizzard contents were not examined due to differential rates of digestion [39]. We considered only animal food items because they are the primary prey of shorebirds and their abundance may influence shorebird distributions (e.g., [12], [16]). Esophageal and proventricular contents were combined and rinsed through a #35 (500 µm) mesh sieve to remove substrate and formalin. Core samples were processed similarly, except samples with a large number (>200) of a single invertebrate taxa were occasionally sub-sampled (up to ¼) using a Folsom plankton splitter. We sorted all items remaining in sieves under dissecting microscopes and classified invertebrate food items to Family or the lowest practical taxonomic level (e.g., Oligochaeta) following Merritt and Cummins [40] and Smith [41]. Individual taxa from each sample were dried to constant mass at 60°C and weighed on a digital balance (±0.1 mg).

Most invertebrates found in diet and core samples were small enough that several individuals of particular taxon were required to measure dry mass. Because many taxa were too small and encountered too infrequently to weigh, we computed an average mass per individual in each taxon and multiplied it by the number of food items not weighed in such instances. Average masses were calculated using the most similar dataset (e.g., same shorebird species’ diet), but became more coarse as taxa became increasingly rare (e.g., all samples within a year).

We converted dry mass (measured or estimated) of important food items to aggregate percent dry mass (hereafter, dry mass) for each shorebird or core sample [42]. We also calculated percent occurrence of food items, and constrained our analyses to items with ≥5% frequency of occurrence for a given shorebird species and year. This strategy eliminated food items that occurred in single individuals and greatly reduced the number of zeros in our dataset.

### Statistical Procedures

#### Fat scoring and size-corrected body mass

We summarized annual MAPS scores of body fat using the MEANS procedure in SAS v9.2, and inferred interannual differences if 95% confidence intervals of mean MAPS scores did not overlap. We used the following morphometrics to compute body mass of shorebirds: 1) head length (±0.1 mm); 2) culmen length (±0.1 mm); 3) tarsus length (±0.1 mm); 4) keel length (±0.1 mm), and; 5) wing-cord length (±1 mm). First, we conducted a principal-components analysis of all morphometric measurements using the PRINCOMP procedure in SAS v9.2 [43], [44]. Then, we included the scores from the first principal component as a covariate in an analysis of covariance for each species (separate analyses) using the MIXED procedure, where body mass was the dependent variable. This allowed us to estimate least-squared means of body mass, which accounted for variation in structural size based on morphometrics (i.e., body mass); we used these least-squares means as our index of body condition. As with fat scoring, we used 95% confidence intervals about averaged body mass estimates to interpret interannual differences.

#### Diet, food abundance, and selection

We attempted to analyze dry mass of important invertebrate taxa found in shorebird diets, collection-site, and random core samples; however, diet proportions were not independent due to the unit-sum constraint (i.e., all proportions of each food item from an individual will sum to 1 and are not independent). Other studies have used compositional analysis to account for this lack of independence [45], but our data set contained many zeros, and this approach may have led to severely inflated Type I error rates [46], [47]. Examination of residual plots indicated our errors were not multivariate-normal distributed and arcsine square-root transforming the data did not significantly improve error distributions and complicated interpretability. Therefore, we followed the approach of other avian diet studies and used species- and year-specific multivariate analysis of variance with proportional dry mass as the dependent variable [47]–[49]. This approach allowed us to evaluate overall variation in important invertebrate taxa (i.e., ≥5% occurrence; dependent variables) found in ingesta, collection-site and random cores for each shorebird species.

We conducted analyses using the MANOVA statement in PROC GLM, SAS v9.2, and included wetland location as a random effect to account for dependence among characteristics within individual wetlands [44]. We used Wilk’s Lambda to evaluate statistical significance of each MANOVA because it is considered robust to violations of the assumption of multivariate normality [45]. If results indicated a significant (*P≤*0.05) difference in composition of invertebrate taxa, we conducted Tukey-Kramer *post-hoc* comparison tests of least-squares means using the PDIFF option of the LSMEANS statement (*P≤*0.05). Although contrasts were performed on least-squares means, we present arithmetic means in tables and text for easier interpretation. Finally, we interpreted results of pairwise contrasts similar to Johnson [31]. That is, we considered comparisons between collection-site and random core samples to be relevant to third-order selection (i.e., selection of specific foraging sites), whereas we considered comparisons of contents of ingesta and collection-site cores relevant to fourth-order selection (i.e., procurement of specific resources; [31], [50]).

We converted dry mass estimates in random core samples to kg/ha and used these data to estimate the average biomass of invertebrate foods found in random samples annually and overall. Biomass estimates are presented ±1 SE and with 95% confidence intervals.

## Results

### Body Condition

Shorebirds that sustained damage to body parts during collection were excluded from analyses of body mass; therefore, we included 149 shorebirds from 4 wetlands in 2007 and 131 shorebirds from 6 wetlands in 2008 in analyses of body condition (Table 1). Estimated body mass of our focal species were within or above reported ranges (Table 2). Estimates of body mass for Least Sandpipers and Lesser Yellowlegs were not different between years (Table 2). Killdeer body mass was 4.2% less in 2008 than 2007, whereas Pectoral Sandpiper body mass was 13% greater in 2008 than in 2007 (Table 2).

**Table 2:** Mean MAPS score (0–7), size-corrected body mass (SCBM; grams), standard error (SE), and 95% lower and upper confidence limits (LCL and UCL) of shorebirds collected in central Illinois during fall migrations 2007 and 2008, and published mass ranges (grams) for target species.

			SCBM				Mass Range^1, 2^	
Year	Species	MAPS	Mass	SE	LCL	UCL	Lower	Upper
2007	Killdeer	0.5	92.5	1.0	90.5	94.5	65	128
	Least Sandpiper	3.5	27.0	0.6	25.8	28.2	9	36
	Lesser Yellowlegs	4.4	113.3	3.4	106.4	130.1	48	114
	Pectoral Sandpiper	4.6	91.5	2.2	87.1	96.0	50	117
2008	Killdeer	1.2	88.6	0.9	86.7	90.4	65	128
	Least Sandpiper	4.9	28.4	0.9	26.5	30.2	9	36
	Lesser Yellowlegs	4.9	112.2	3.5	105.1	119.3	48	114
	Pectoral Sandpiper	5.9	102.9	2.5	97.9	107.9	50	117

1 Poole, A. ed. (2005) The Birds of North America Online: http://bna.birds.cornell.edu/BNA/. Cornell Laboratory of Ornithology, Ithaca, NY.

2 O’Brien M, Crossley R, and Karlson K (2006) The Shorebird Guide. Houghton Mifflin Company. New York, USA.

### Diet and Food Availability

#### Killdeer

We collected 35 Killdeer from 4 wetlands in 2007 and 34 Killdeer from 5 wetlands in 2008. Of these, 27 (77%) from 2007 and 18 (53%) from 2008 contained adequate amounts of invertebrate food items (i.e., percent occurrence ≥5%) for analyses. We identified 13 taxa in Killdeer diets in 2007 and 6 taxa in 2008 (14 total; *see* Table S1 for complete taxa list). Coleoptera (36.7% [2007], 16.7% [2008]), Diptera (36.7% [2007], 22.2% [2008]), and Nematoda (66.7% [2007], 55.6% [2008]) occurred most frequently in Killdeer diets in each year. Dry mass of invertebrate taxa differed among Killdeer diets, collection-site and random core samples in 2007 (Wilks’ λ = 0.15; *F*_22, 130_ = 9.42, *P*<0.001) and 2008 (Wilks’ λ = 0.26; *F*_22, 74_ = 3.20, *P*<0.001).

#### Foraging site and diet selection

Only dry mass of Ostracoda in 2007 was significantly different between collection (5%) and random sites (0%; Table 3), suggesting there was little support for third-order (foraging site) selection by Killdeer (Table 3). However, significant differences in dry mass of invertebrate Orders found in Killdeer diets and collection-site core samples indicated active selection or avoidance of some diet items (i.e., fourth-order selection occurred; Table 3). In both years, Killdeer consumed significantly more Nematoda than were found in collection site samples, and significantly fewer Oligochaetes compared with their high dry mass at collection sites (Table 3). In 2007, Killdeer diets contained significantly greater dry mass of Coleoptera than were found at collection sites; this trend was also present in 2008, but was not statistically significant. Finally, Killdeer consumed significantly less Ostracoda in 2007 than were present in collection site samples, although this difference (4% dry mass) was relatively small.

**Table 3:** Aggregate percent mass (dry) of taxa found in fall-migrating Killdeer ingesta and core samples taken at collection and random sites in 2007 and 2008.

	2007						2008					
Taxa	Diet		Collection		Random		Diet		Collection		Random	
Amphipoda	.		.		.		0	A	0.3	A	0	A
Bivalvia	0	A	0.7	A	4.9	A	.		.		.	
Cladocera	.		.		.		0	A	2	A	0	A
Coleoptera	33	A	3.5	B	10.3	B	16.7	A	8.1	A	26.9	A
Diptera	24.7	A	15.1	A	12	A	22.2	A	27.2	A	13.8	A
Ephemeroptera	0	A	0.3	A	0	A	.		.		.	
Fish	.		.		.		0	A	0	A	T	A
Gastropoda	0	A	7	AB	13.8	B	0	A	10.7	AB	20.3	B
Hemiptera	9.1	A	3.7	A	0.4	A	0	A	0.1	A	2.6	A
Hirudinea	6.7	A	1.5	A	1.8	A	16.3	A	0	A	5.4	A
Isopoda	0		2.3		0		0		0.2		0	
Nematoda	20.2	A	1.2	B	1.2	B	39.3	A	0.4	B	1.7	B
Oligochaeta	0.9	A	59.5	B	51.5	B	5.6	A	42.2	B	28.1	B
Ostracoda	1	A	5	B	0	A	0	A	0.1	A	0	A
Trichoptera	4.3	A	0.2	A	4.1	A	0	A	8.8	A	1.3	A

* Values with different letters within rows indicate significant differences of least-squares means (Tukey-Kramer test: *P*≤0.05) within that year.

T indicates a trace amount of material present.

### Least Sandpiper

We collected 36 Least Sandpipers from 3 wetlands in 2007 and 29 Least Sandpipers from 5 wetlands in 2008. Of these, 30 (83%) from 2007 and 17 (59%) from 2008 contained adequate food in the upper digestive tract for analyses. Least Sandpipers consumed 9 taxa in 2007 and 5 taxa in 2008 (10 total; *see* Table S2 for complete taxa list). Diptera (70.0% [2007], 77.8% [2008]) and Coleoptera (30.0% [2007], 11.1% [2008]) were the most common taxa consumed in each year. Dry mass of invertebrate taxa differed among Least Sandpiper diets, collection-site core samples, and random core samples in 2007 (Wilks’ λ = 0.22; *F*_22, 150_ = 7.77, *P*<0.001) and 2008 (Wilks’ λ = 0.19; *F*_22, 70_ = 4.14, *P*<0.001).

#### Foraging site and diet selection

There was little support for third-order selection by Least Sandpipers (Table 4). Statistically more Nematoda were found in collection-site than random core samples in 2007, although the mean difference was only 0.7% dry mass. In 2008, dry mass of Oligochaeta was significantly greater in collection-site than random samples, but both estimates were relatively great. Significant differences in dry mass of invertebrate taxa found in Least Sandpiper diets and collection-site core samples indicated that fourth-order selection occurred (Table 4). In both years, contrasts of least-squares means indicated that Least Sandpipers avoided consuming Oligochaeta, but selected Diptera (Table 4). In 2007, Least Sandpipers consumed fewer Ostracoda and Nematoda than were found in collection-site samples, though both mean differences were relatively small (1.0–5.2% dry mass; Table 4).

**Table 4:** Aggregate percent mass (dry) of taxa found in fall-migrating Least Sandpiper ingesta and core samples taken at collection and random sites in 2007 and 2008.

	2007						2008					
Taxa	Diet		Collection		Random		Diet		Collection		Random	
Arachnida	0	A	0	A	2.9	A	.		.		.	
Bivalvia	0	A	2.1	A	1.1	A	0	A	0	A	0.4	A
Cladocera	.		.		.		0	A	T	A	0	A
Coleoptera	28	A	6.2	B	7.8	B	11.8	A	8.8	A	1.8	A
Diptera	63.7	A	15.5	B	19.4	B	82.4	A	17.1	B	36.3	B
Gastropoda	0	A	2.9	A	1.8	A	0	A	0	A	2.1	A
Hemiptera	0.3	A	1.6	A	0.9	A	0	A	0	A	4	A
Hirudinea	4.5	A	3.6	A	2.6	A	0	A	0	A	3.7	A
Isopoda	0	A	1.1	A	0	A	0	A	0.2	A	4.1	A
Nematoda	0	A	1	B	0.3	A	0	A	0.6	A	6.1	A
Oligochaeta	3.5	A	60.2	B	59.3	B	5.9	A	72.7	B	41.5	C
Ostracoda	0.3	A	5.5	B	0.3	A	0	A	T	A	0	A
Trichoptera	0	A	0.4	A	3.5	A	0	A	0.5	A	0	A

* Values with different letters within rows indicate significant differences of least-squares means (Tukey-Kramer test: *P*≤0.05) within that year.

T indicates a trace amount of material present.

### Lesser Yellowlegs

We collected 39 Lesser Yellowlegs from 3 wetlands in 2007, of which 34 (87%) contained food in the upper digestive tract. In 2008, we collected 32 Lesser Yellowlegs from 4 wetlands, of which 20 (63%) contained food in the upper digestive tract. We identified 11 invertebrate taxa in Lesser Yellowlegs diets in 2007 and 9 in 2008 (15 total; *see* Table S3 for complete taxa list). Diptera were the most important food by percent occurrence (33.3% [2007], 40.0% [2008]) and dry mass in both years (Table 5). Coleoptera occurred relatively frequently in 2007 (25.0%) but not in 2008 (5.0%), whereas the converse was true for fishes (*Gambusia* sp.; absent in 2007, 20.0% in 2008). Dry mass of invertebrate taxa differed among Lesser Yellowlegs diets, collection-site, and random core samples in 2007 (Wilks’ λ = 0.24; *F*_24, 172_ = 7.39, *P*<0.001) and 2008 (Wilks’ λ = 0.25; *F*_24, 88_ = 3.60, *P*<0.001).

**Table 5:** Aggregate percent mass (dry) of taxa found in fall-migrating Lesser Yellowlegs ingesta and core samples taken at collection and random sites in 2007 and 2008.

	2007						2008					
Taxa	Diet		Collection		Random		Diet		Collection		Random	
Bivalvia	0	A	0.6	A	0.4	A	4.2	A	0	A	2.1	A
Cladocera	0	A	T	A	0	A	0	A	0.1	A	0	A
Coleoptera	23.7	A	0.4	B	3.2	B	0.3	A	0	A	0	A
Diptera	24.4	A	33.5	A	21.9	A	31.4	A	29.6	A	34	A
Ephemeroptera	2.7	A	0	A	0	A	.		.		.	
Fish	.		.		.		19.6	A	0	B	0	B
Gastropoda	0	A	5.7	A	5.6	A	0	A	0	A	1	A
Hemiptera	18.9	A	3.4	B	3.2	B	12.8	A	0.1	A	4.3	A
Hirudinea	4	A	5.4	A	9.1	A	0	A	4.4	A	3.7	A
Isopoda	0	A	0	A	0.1	A	1.6	A	1.4	A	1.1	A
Nematoda	3.6	A	3	A	1.1	A	22.5	A	0.5	B	0.4	B
Oligochaeta	0	A	46.8	B	53.4	B	5	A	57.3	B	53.3	B
Ostracoda	13.4	A	0.2	B	0.2	B	2.6	A	0	A	0	A
Trichoptera	9.5	A	1	A	1.8	A	0	A	6.5	A	0.2	A

* Values with different letters within rows indicate significant differences of least-squares means (Tukey-Kramer test: *P*≤0.05) within that year.

T indicates a trace amount of material present.

#### Foraging site and diet selection

Pairwise comparisons revealed no differences in dry mass of invertebrate taxa found in collection and random site core samples in 2007 or 2008, indicating no support for third-order selection (Table 5). However, multiple comparison of dry mass of invertebrate taxa between collection sites and diets supported fourth-order selection (Table 5). Lesser Yellowlegs clearly avoided Oligochaeta in both years, whereas they contained greater dry mass of Hemiptera, Ostracoda and Coleoptera than found in collection-site cores, although the differences were only significant in 2007 (Table 5). In 2008, Lesser Yellowlegs diets contained significantly more Nematoda and fish (*Gambusia* sp.) than found in collection-site samples, though the latter diet items were attributable to 4 individuals that had relatively great dry masses and our sampling strategy was not designed to capture vertebrates.

### Pectoral Sandpiper

We collected 39 Pectoral Sandpipers from 3 wetlands in 2007 and 36 from 3 wetlands in 2008, of which 37 (95%) and 28 (78%), respectively, contained food in the upper digestive tract. We found 9 invertebrate taxa in Pectoral Sandpiper diets in 2007 and 7 in 2008, respectively (13 total; *see* Table S4 for complete taxa list). Diptera, specifically Chironomidae, were the predominant food by percent occurrence (73.7% [2007], 71.4% [2008]) and dry mass (Table 6) in both years. Dry mass of invertebrate taxa differed among Pectoral Sandpiper diets, collection-site core samples, and core samples taken at random locations in 2007 (Wilks’ λ = 0.36; *F*_22, 192_ = 5.80, *P*<0.001) and 2008 (Wilks’ λ = 0.17; *F*_22, 136_ = 8.97, *P*<0.001).

**Table 6:** Aggregate percent mass (dry) of taxa found in fall-migrating Pectoral Sandpiper ingesta and core samples taken at collection and random sites in 2007 and 2008.

	2007						2008					
Taxa	Diet		Collection		Random		Diet		Collection		Random	
Arachnida	0	A	T	A	0	A	0	A	0	A	0.1	A
Bivalvia	0	A	3	A	2.6	A	3.4	A	3.6	A	0.9	A
Cladocera	.		.		.		0	A	T	A	0	A
Coleoptera	3.7	A	0.4	A	4.2	A	0	A	0.5	A	0	A
Diptera	67.1	A	38.3	B	30.7	B	72.9	A	24.3	B	21.7	B
Gastropoda	2.7	A	5.5	A	2.2	A	0	A	1.5	A	0.4	A
Hemiptera	13.6	A	3.8	B	0.9	B	0	A	0.1	AB	0.7	B
Hirudinea	0	A	3.7	A	5.2	A	0	A	0.6	A	0	A
Isopoda	0	A	0	A	0.2	A	19.8	A	2.5	B	4.1	B
Nematoda	7.2	A	1.7	A	2.6	A	3.8	A	0.5	A	0.7	A
Oligochaeta	1.5	A	41.1	B	50.5	B	0	A	65.9	B	71.5	B
Ostracoda	2.7	A	0.8	A	T	A	0	A	T	A	0	A
Trichoptera	1.5	A	1.8	A	0.8	A	0	A	0.7	A	0	A

* Values with different letters within rows indicate significant differences of least-squares means (Tukey-Kramer test: *P*≤0.05) within that year.

T indicates a trace amount of material present.

#### Foraging site and diet selection

Pairwise comparisons revealed no differences in dry mass of invertebrate taxa found in collection-site and random core samples for Pectoral Sandpipers in 2007 or 2008, indicating no support for third-order selection (Table 6). Conversely, pairwise comparisons of dry mass of invertebrate taxa supported fourth-order selection by Pectoral Sandpipers in 2007 and 2008 (Table 6). As with the other 3 species we collected, Pectoral Sandpipers avoided Oligochaetes in both years, whereas dry mass of Diptera was greater in collection-site samples in both years. Some year-specific differences in Pectoral Sandpiper diets also existed. In 2007, diets contained significantly more Hemiptera than collection-site samples, whereas selection for Isopoda at foraging sites occurred in 2008 (Table 6).

### Invertebrate Biomass

Estimated biomass of invertebrates found in core samples collected at random was 47.0±4.3 (SE) kg/ha (dry mass; 95% CI: 38.5–55.5) in 2007 and 56.0±8.1 (SE) kg/ha (dry mass; 95% CI: 40.0–72.1) in 2008. Confidence intervals about annual invertebrate biomass estimates overlapped considerably; thus, estimated average biomass across all years and sites was 51.2±4.4 (SE) kg/ha (dry mass; 95% CI: 42.5–59.9).

## Discussion

### Foraging Site and Diet Selection

#### Third-order selection

Our results provided sparse evidence for third-order, or habitat patch selection [31] of feeding sites by fall-migrating shorebirds in the IRV (Tables 3, 4, 5, and 6). However, we did find support for selection of specific food items (i.e., fourth-order selection [31]) and consistent avoidance of Oligochaeta among our focal shorebird species. Although relatively few studies have examined selection among diet, foraging site, and random sites, our results contrast others who suggest that shorebirds may select foraging sites based on food abundance [16], [26], [32], [51], [52]. Previous studies have documented selection of foraging sites by shorebirds; however, many of these used sampling designs that constrained inference or resulted in multiple interpretations (e.g., [12], [20], [53]). In contrast, by comparing random and foraging site samples we found little support for third-order selection by shorebirds. We perceived individual wetlands in our study to be relatively homogeneous in terms of mudflat habitat and invertebrate availability, but nonetheless we suspected that proximate cues, such as micro-topography or perceived predation risk, might have allowed shorebirds to select foraging sites that were more profitable than expected at random [52]. Further, we acknowledge that sample sizes may have been too small in some instances to detect differences if they existed. For example, although not statistically different, dry mass of Coleoptera was 232% greater in random samples (26.9%) than collection-site samples (8.1%) taken for Killdeer in 2008. Nonetheless, most differences between collection and random site dry mass were inconsistent among shorebird species and years, and were small in comparison to the differences between dry mass of diets and collection site samples.

#### Fourth-order selection

In addition to studies of third-order foraging habitat selection in shorebirds, several studies have evaluated selection of diet items at collection sites (i.e., fourth-order selection; [31]); [12], [13], [24], [32], [54], [55]. Our results indicated selection of specific invertebrate taxa by four study species, which contrasts results of some published research [12], [13], [24], [54], but supports others [32], [55]. Previous studies reported that shorebirds typically consume prey opportunistically with little relation to nutritional or energetic value [12], [13]. An opportunistic approach and flexible, compositional diet, theoretically allows shorebirds to consume a variety of prey in the highly variable wetland habitats of North America [11], [17]. Similar to studies of third-order selection, many previous studies had constraints that may have limited inference, such as insufficient invertebrate sampling and diet preservation [54], sampling of invertebrates that may have been physically unavailable to foraging shorebirds [13], and generalized analyses and summarization of diet contents [24]. Our results provide particularly strong evidence for selection of Diptera (Pectoral and Least Sandpipers), Coleoptera (Least Sandpipers and Lesser Yellowlegs), Nematoda (Killdeer), and Hemiptera (Pectoral Sandpipers and Lesser Yellowlegs).

Several possible mechanisms could explain the selective foraging observed in our study, and each may have implications for conservation planning and habitat management. First, we documented considerably greater invertebrate biomass at foraging sites compared with other shorebird food selection studies. Previous research has reported that the availability of benthic invertebrates (dry biomass) varies dramatically among wetland systems and seasons. Estimates range from very low (e.g., 1.8–9.2 kg/ha dry biomass; [12], [13], [56], [57]) to extremely high (e.g., 278.2 kg/ha dry biomass; [58]), and many values in between [59]–[61]. Our overall estimate of invertebrate biomass (51.2 kg/ha) represents a value closer to the median of the biomass range and is similar to other estimates from nearby wetlands in the IRV and Mississippi River [62]–[64]. In contrast, most previous shorebird food selection studies reported substantially lower invertebrate biomasses, and indicated that shorebirds in those areas foraged opportunistically [12], [13], [56], [57]. Optimal foraging theory generally predicts that absolute abundance of potential food items (controlling for handling time; i.e., equal availability of different food types) influences dietary specialization [65], [66]. Specifically, as total food abundance increases, foragers should increase selectivity to where, eventually, only one prey type might be consumed even if all were equally available [66]. We suggest that the selective foraging we observed may be a function of absolute abundance and biomass of invertebrate foods, and similar research in other high-biomass habitats may yield similar results [32].

Another potential explanation for the diet selectivity we observed may relate to the condition of birds in our study. The majority of shorebirds we collected were in good to excellent body condition (Table 2); however, we do not know how long shorebirds were present at our study sites prior to collection. It is well established that diet affects body condition, but body condition may in-turn dictate diet [67]. Thus, if shorebirds arrived at our study area in good condition, they may have been more selective in their diet. In contrast, migrants arriving in poorer condition may be more likely to consume food opportunistically to quickly improve condition. Other researchers have suggested the opposite, whereby birds in better condition forage opportunistically, and those in poor condition seek out higher quality foods [68], [69]. Clearly, relationships between body condition and diet may be complex, and more explicit research is needed to clarify these relationships. Understanding such relationships may yield important implications for management, such as quantifying the true value of foraging habitats in our study region compared to other stopover sites.

Finally, the diet selectivity we documented may have been related to the composition of invertebrate foods at our study sites. Past results of shorebird diet studies were likely biased towards invertebrates with hard body parts, because soft-bodied invertebrates may have been lost or degraded due to post-mortem digestion if not properly preserved [26], [39], [70]. Many taxa in our study were considered soft-bodied, and if these invertebrates were consumed but not preserved prior to digestion it is possible our analyses would indicate avoidance of these taxa. Oligochaetes were the primary soft-bodied taxa that were consumed considerably less than found in collection-site samples, which would not have been predicted if shorebirds foraged opportunistically. Other researchers have suggested that Oligochaetes may be underrepresented in waterbird diets because of their fragility and small size [26], [53], [71]. Further, it is difficult to imagine a functional reason for avoidance of Oligochaetes. For example, gross energy and crude protein of Oligochaetes is similar to, or greater than that of Chironomidae [60], which are readily consumed by many waterbird species. We do not believe the apparent avoidance of Oligochaetes in our study was a function of methodology. We were keenly aware of potential post-mortem digestion of soft-bodied invertebrates and irrigated the upper digestive tract of each shorebird with a formalin solution as quickly as possible, typically within 1–2 minutes of collection. Additionally, Oligochaetes were common in diets but greatly underrepresented in aggregate percent dry mass, precluding the possibility that they were missed entirely. Thus, we contend avoidance of Oligochaetes in our study was a real phenomenon.

Other researchers have also reported apparent avoidance or lack of consumption of Oligochaetes by shorebirds [25], [56], [60], [71], [72] and other waterbirds [73], despite the fact they are often the most abundant taxa in benthic substrates. Indeed, growing evidence suggests that waterbirds consume Oligochaetes less than their availability. One possibility for this is that shorebirds do not actively avoid Oligochaetes, but rather Oligochaetes are able to avoid foraging shorebirds [74], [75], either by moving away from the forager or by avoiding detection [76]. Predator avoidance in invertebrates is not universal; it occurs in some taxa [77]–[79] but not in others [80]. However, Oligochaetes have the ability to migrate in response to chemical (dissolved oxygen, [75]) or physical (drying, [74]) stimuli. Thus, it seems plausible that Oligochaetes might be able to detect the presence of predators moving near them (e.g., pressure) and migrate away from the surface. Additionally, some shorebirds forage using fine sensory mechanoreceptors in their bill-tips, which are capable of detecting small vibrations created by buried invertebrates [76], or through some other form of remote sensing [78], [81]. Perhaps Oligochaetes avoid these forms of detection, and are functionally undetectable to foraging shorebirds except when encountered tactilely. Finally, Oligochaetes may be associated with plant roots and other organic material, which could make them difficult to exploit [72]. Oligochaetes at our IRV sites appeared to be widely and relatively homogeneously distributed, although we did not specifically explore subsurface associations. Additional research examining the role of Oligochaetes as food in various wetland systems with inconsistent invertebrate biomass is warranted.

#### Body condition

Results of size corrected body mass support the notion that our study wetlands in the IRV provided high-quality foraging habitats for shorebirds, given that Killdeer, Least and Pectoral Sandpipers, and Lesser Yellowlegs were in good to excellent body condition during our study. Mass of Killdeer varies considerably (65–128 g, [82], [83]), but our 2007 and 2008 body mass estimates (Table 2) were within the reported range. Body mass of Least Sandpipers collected in 2007 and 2008 (Table 2) were near the upper range of reported body mass ([83], [84]; 9–36 g). Lesser Yellowlegs’ body mass (Table 2) in both years was greater than average masses reported by Tibbitts and Moskoff ([85]; 67–94 g), but at the upper extent of that reported by O’Brien et al. ([83]; 48–114). Pectoral Sandpiper body mass (Table 2) in 2007 and 2008 was also within the reported range of body masses (50–117 g, [6], [83]). Killdeer and Pectoral Sandpiper body mass differed between years (body mass was higher for Killdeer in 2007 and Pectoral Sandpiper in 2008) which may be associated with different habitat conditions. We speculate that specific habitat conditions created by variation in timing, duration, and intensity of spring and summer flooding within the IRV may have created drier conditions that favored Killdeer in 2007 [5], [82], whereas wetter conditions may have favored Pectoral Sandpipers in 2008 [18].

If shorebirds acquired fat resources at our study wetlands, the magnitude of accumulation would have been somewhat dependent on the time they spent at our study site. Thomas [86] reported that Least and Pectoral Sandpipers arrived at stopover locations with excess fat stores, and fat stores and body condition were not significant predictors of stopover duration. We were unable to evaluate stopover duration of shorebirds in our study, but suggest that high food abundance, coupled with the fact that some shorebird species can increase body mass by 70% or more at migratory stopovers [87], supports the notion that fat stores were gained at our study wetlands. Since fat stores acquired before migration can have a pronounced impact on survival [3], fat acquisition at our study area would be indicative of high-quality foraging habitat.

#### Prey depletion

We did not specifically address prey depletion during our study, but several other authors have suggested shorebird foraging can substantially reduce prey abundance over the course of migration [10], [12], [26]. Although we found sparse evidence for selection of specific foraging sites during our study, it is possible those quantitative and qualitative differences in dry mass between foraging and random sites were due to depletion. However, the relative abundance of shorebird prey items and high body condition of birds in our study suggested that adequate food was acquired quickly before birds moved on (e.g., a short-hop strategy; [26], [88]) and depletion was not significant.

### Conservation and Management Implications

A primary goal of shorebird conservation is to provide and maintain adequate carrying capacity (in terms of energy) to support migrating shorebirds and meet regional population objectives, which are based on proportions of species-specific objectives under the continental shorebird plan [5∶24]. To meet these goals, habitat objectives must be met in target areas relative to population estimates. Most continental and regional estimates of shorebird population sizes are tenuous; therefore, it is difficult to provide precise and targeted recommendations regarding habitat availability and abundance. Despite these uncertainties, functional habitat is essential to support migrating shorebirds in mid-continent areas. We propose our results of relatively high invertebrate biomass, diet selectivity, and generally good to excellent body condition demonstrate that when shorebird foraging habitat is available in the IRV (and perhaps other bottomland wetlands in the region) it is not only functional, but likely of high quality.

Safran et al. [53] proposed that suitable water level may be a more important determinant of foraging habitat selection by shorebirds than food abundance or availability of specific foods. To this end, most wetlands in the IRV (both publicly- and privately-owned) are dewatered annually during mid-summer (i.e., moist-soil management) which results in water levels that provide extensive foraging habitat for migrating shorebirds. Our research concurs with the body of literature that suggests these draw-downs often provide expansive mud flats for foraging shorebirds [32], [36], [71], [89], even though management for shorebirds is not likely a goal of private or some public land managers. However, abundance and availability of shorebird foraging habitat in the IRV can be incredibly variable due to the dynamic and altered hydrology of the Illinois River [33], [35]. Indeed, foraging habitat may vary within a season from >20 large (e.g., >100 ha) dewatered wetland basins in La Grange Pool with expansive mudflats, to virtually no foraging habitat for shorebirds in a matter of days following substantial upstream precipitation events (e.g., ≥5 cm of rain). Such expansive flooding during late-spring through summer prevents managers from dewatering wetlands, which effectively eliminates all shorebird foraging habitats in the IRV. Thus, the current hydrology of the Illinois River frequently results in an “all or nothing” scenario for shorebird foraging habitat.

Most previous shorebird studies have been conducted on public lands, but our study included several private wetlands that were in close proximity to publicly-managed sites of known importance to migrating shorebirds. It is likely that some, or even most, of these private wetlands have not been previously surveyed for shorebirds. Although we did not record shorebird abundances, substantial numbers of shorebirds used private wetlands in our study area, indicating greater shorebird abundance in the region than previously reported, including species that the JV identifies as having population deficits during migration [5]. We speculate that at least some of this deficit may be due to a lack of survey data for, or access to, lesser-known stopover areas, especially in the interior of the continent, but surveys of these areas may provide critical information to refine, and perhaps even reduce, population and habitat objectives [5].

Our results indicated that foraging shorebirds avoided Oligochaetes, and if this taxon is truly avoided or unavailable to fall migrating shorebirds, they should not be considered in estimates of forage biomass. In this scenario, our overall biomass estimate would be reduced by 51% to 25.0 kg/ha. We note, however, that each of our 4 focal shorebird species consumed Oligochaetes in at least 1 year (Tables 3, 4, 5, and 6), indicating that, although not preferred, foraging shorebirds will, at least occasionally, consume this common invertebrate. Further, other researchers have reported Oligochaetes in shorebird diets [24], [26], or that they were considered important shorebird foods [57], [90]. Thus, it is likely not appropriate to dismiss Oligochaetes as food items, but research to understand the relationship between shorebird foraging and Oligochaete abundance, behavior, distribution (including vertical), and microhabitat associations would enhance our understanding of food availability and, hence, carrying capacity for migrating shorebirds. We recommend targeted investigations that focus on relationships between abundance, movements, and spatial distributions of Oligochaetes and other wetland taxa in relation to shorebird foraging ecology. Such studies might be best accomplished through controlled experiments [76], [78], [81].

Our biomass estimates originated from backwater wetlands of a large inland river system in the Midwestern United States, and our study wetlands had similar management strategies and histories. Thus, it may be inappropriate to apply our estimates of invertebrate biomass to other regions or drastically different aquatic systems. Conversely, similar estimates exist for backwater wetlands of the Illinois and Mississippi River systems within and near our study region [26], [63], [64]. Thus, we believe that wetlands associated with large river systems in the mid-continental United States may support appreciably greater invertebrate biomasses than isolated palustrine or lacustrine wetlands [91]. Therefore, these wetlands associated with large rivers have greater carrying capacity, and the ability to support substantial numbers of shorebirds during migration periods. Spatially clustered wetlands that form complexes, similar to those in the IRV, may be perceived by migrating shorebirds as single, large wetlands [92], thereby increasing their attractiveness over individual wetlands [57], [93]. Consequently, we suggest habitat creation, improvement, or protection focused within floodplains of large river corridors [94], with special consideration given to wetlands with flood protection. This focus would promote wetlands that avoid complete inundation (i.e., habitat loss) during critical times of the year, as shorebirds have few mid-continent options for migration habitat [86]. We suggest that these wetlands will provide greater conservation value as opposed to individual wetlands or complexes with lower invertebrate biomass potential [92]. Although our study should be replicated in other locations with high invertebrate biomass, we advise that focusing limited conservation resources on such habitats will provide greater habitat value than could be achieved by non-targeted conservation actions [5]. Such actions may include creation, enhancement, or protection of shorebird habitat isolated from traditional stopover locations or with low invertebrate biomass potential.

## Supporting Information

Table S1 **Aggregate percent mass (dry) of taxa found in fall migrating Killdeer ingesta and core samples taken at collection and random sites in 2007 (** ***n*** **= 27) and 2008 (** ***n*** **= 18).** Values with different letters within Taxa Orders (rows) indicate significant differences of least-squares means (Tukey-Kramer test: *P*≤0.05).
(DOCX)

Table S2 **Aggregate percent mass (dry) of taxa found in fall migrating Least Sandpiper ingesta and core samples taken at collection and random sites in 2007 (** ***n*** **= 30) and 2008 (** ***n*** **= 17).** Values with different letters within Taxa Orders (rows) indicate significant differences of least-squares means (Tukey-Kramer test: *P*≤0.05).
(DOCX)

Table S3 **Aggregate percent mass (dry) of taxa found in fall migrating Lesser Yellowlegs ingesta and core samples taken at collection and random sites in 2007 (** ***n*** **= 34) and 2008 (** ***n*** **= 20).** Values with different letters within Taxa Orders (rows) indicate significant differences of least-squares means (Tukey-Kramer test: *P*≤0.05).
(DOCX)

Table S4 **Aggregate percent mass (dry) of taxa found in Pectoral Sandpiper ingesta and core samples taken at collection and random sites in 2007 (** ***n*** **= 37) and 2008 (** ***n*** **= 28).** Values with different letters within Taxa Orders (rows) indicate significant differences of least-squares means (Tukey-Kramer test: *P*≤0.05).
(DOCX)
