# Supplementary material for: Foraging Ecology of Fall-Migrating Shorebirds in the Illinois River Valley
Source: PLoS One. 2012 Sep 18;7(9):e45121. doi: 10.1371/journal.pone.0045121 (PMC3445572; doi:10.1371/journal.pone.0045121)
Supplement: Table S1 — Aggregate percent mass (dry) of taxa found in fall migrating Killdeer ingesta and core samples taken at collection and random sites in 2007 ( n = 27) and 2008 ( n = 18). Values with different letters within Taxa Orders (rows) indicate significant differences of least-squares means (Tukey-Kramer test: P≤0.05). (DOCX) [file pone.0045121.s001.docx]

Table S1.

|  |  |  |  |  | |  | |  |  |  | |  |  | |  | |  | |
| --- | --- | --- | --- | --- | --- | --- | --- | --- | --- | --- | --- | --- | --- | --- | --- | --- | --- | --- |
|  | 2007 | | | | | | | | 2008 | | | | | | | | | |
| Taxa | Diet | | Collection | | | | Random | | Diet | | | Collection | | | | Random | | |
| **Amphipoda** | . |  | . | |  | | . |  | 0.0 | | A | 0.3 | | A | | 0.0 | | A |
| **Bivalvia** | 0.0 | A | 0.7 | | A | | 4.9 | A | . | |  | . | |  | | . | |  |
| Sphaeriidae | 0.0 |  | 0.7 | |  | | 4.9 |  | . | |  | . | |  | | . | |  |
| **Cladocera** | . |  | . | |  | | . |  | 0.0 | | A | 2.0 | | A | | 0.0 | | A |
| **Coleoptera** | 33.0 | A | 3.5 | | B | | 10.3 | B | 16.7 | | A | 8.1 | | A | | 26.9 | | A |
| Carabidae | 0.0 |  | 1.4 | |  | | 0.0 |  | . | |  | . | |  | | . | |  |
| Chrysomelidae | 0.0 |  | 0.4 | |  | | 0.0 |  | . | |  | . | |  | | . | |  |
| Curculionidae | 4.3 |  | 0.0 | |  | | 0.0 |  | . | |  | . | |  | | . | |  |
| Dytiscidae | . |  | . | |  | | . |  | 0.0 | |  | 0.7 | |  | | 0.0 | |  |
| Elmidae | 0.0 |  | 0.0 | |  | | 2.0 |  | . | |  | . | |  | | . | |  |
| Heteroceridae | 16.8 |  | 0.0 | |  | | 1.5 |  | 16.7 | |  | 1.9 | |  | | 20.0 | |  |
| Hydrophilidae | 12.0 |  | 1.5 | |  | | 6.8 |  | 0.0 | |  | 2.8 | |  | | 6.9 | |  |
| Staphylinidae | 0.0 |  | 0.1 | |  | | 0.0 |  | 0.0 | |  | 2.7 | |  | | 0.0 | |  |
| **Diptera** | 24.7 | A | 15.1 | | A | | 12.0 | A | 22.2 | | A | 27.2 | | A | | 13.8 | | A |
| Ceratopogonidae | 4.0 |  | 1.8 | |  | | 2.6 |  | 0.0 | |  | 3.2 | |  | | 1.0 | |  |
| Chironomidae | 12.6 |  | 10.5 | |  | | 7.3 |  | 11.1 | |  | 6.8 | |  | | 6.9 | |  |
| Dolichopodidae | 0.2 |  | 0.5 | |  | | 1.4 |  | 0.0 | |  | 3.3 | |  | | 1.0 | |  |
| Empididae | 8.1 |  | 2.1 | |  | | 0.0 |  | 0.0 | |  | 0.8 | |  | | 0.0 | |  |
| Ephydridae | 0.0 |  | 0.1 | |  | | 0.7 |  | 0.0 | |  | 1.2 | |  | | 0.0 | |  |
| Sciomyzidae | 0.0 |  | 0.1 | |  | | 0.0 |  | 0.0 | |  | 6.3 | |  | | 4.7 | |  |
| Stratiomyidae | . |  | . | |  | | . |  | 11.1 | |  | 4.6 | |  | | 0.1 | |  |
| **Ephemeroptera** | 0.0 | A | 0.3 | | A | | 0.0 | A | . | |  | . | |  | | . | |  |
| Baetidae | 0.0 |  | 0.3 | |  | | 0.0 |  | . | |  | . | |  | | . | |  |
| **Fish** | . |  | . | |  | | . |  | 0.0 | | A | 0.0 | | A | | T | | A |
| *Gambusia spp*. | . |  | . | |  | | . |  | 0.0 | |  | 0.0 | |  | | T | |  |
| **Gastropoda** | 0.0 | A | 7.0 | | AB | | 13.8 | B | 0.0 | | A | 10.7 | | AB | | 20.3 | | B |
| Lymnaeidae | . |  | . | |  | | . |  | 0.0 | |  | 1.9 | |  | | 0.8 | |  |
| Physidae | 0.0 |  | 3.5 | |  | | 7.0 |  | 0.0 | |  | 3.8 | |  | | 16.5 | |  |
| Planorbidae | 0.0 |  | 3.5 | |  | | 6.7 |  | 0.0 | |  | 5.0 | |  | | 3.0 | |  |
| **Hemiptera** | 9.1 | A | 3.7 | | A | | 0.4 | A | 0.0 | | A | 0.1 | | A | | 2.6 | | A |
| Corixidae | 9.1 |  | 3.7 | |  | | 0.4 |  | 0.0 | |  | 0.1 | |  | | 2.6 | |  |
| **Hirudinea** | 6.7 | A | 1.5 | | A | | 1.8 | A | 16.3 | | A | 0.0 | | A | | 5.4 | | A |
| Glossiphonidae | 6.7 |  | 1.5 | |  | | 1.8 |  | 16.3 | |  | 0.0 | |  | | 5.4 | |  |
| **Isopoda** | 0.0 |  | 2.3 | |  | | 0.0 |  | 0.0 | |  | 0.2 | |  | | 0.0 | |  |
| **Nematoda** | 20.2 | A | 1.2 | | B | | 1.2 | B | 39.3 | | A | 0.4 | | B | | 1.7 | | B |
| **Oligochaeta** | 0.9 | A | 59.5 | | B | | 51.5 | B | 5.6 | | A | 42.2 | | B | | 28.1 | | B |
| **Ostracoda** | 1.0 | A | 5.0 | | B | | 0.0 | A | 0.0 | | A | 0.1 | | A | | 0.0 | | A |
| **Trichoptera** | 4.3 | A | 0.2 | | A | | 4.1 | A | 0.0 | | A | 8.8 | | A | | 1.3 | | A |
| Leptoceridae | 4.3 |  | 0.2 | |  | | 4.1 |  | 0.0 | |  | 8.8 | |  | | 1.3 | |  |
