# Supplementary material for: Foraging Ecology of Fall-Migrating Shorebirds in the Illinois River Valley
Source: PLoS One. 2012 Sep 18;7(9):e45121. doi: 10.1371/journal.pone.0045121 (PMC3445572; doi:10.1371/journal.pone.0045121)
Supplement: Table S2 — Aggregate percent mass (dry) of taxa found in fall migrating Least Sandpiper ingesta and core samples taken at collection and random sites in 2007 ( n = 30) and 2008 ( n = 17). Values with different letters within Taxa Orders (rows) indicate significant differences of least-squares means (Tukey-Kramer test: P≤0.05). (DOCX) [file pone.0045121.s002.docx]

Table S2.

|  |  |  |  |  |  |  |  |  |  |  |  |  |
| --- | --- | --- | --- | --- | --- | --- | --- | --- | --- | --- | --- | --- |
|  | 2007 | | | | | | 2008 | | | | | |
| Taxa | Diet | | Collection | | Random | | Diet | | Collection | | Random | |
| **Arachnida** | 0.0 | A | 0.0 | A | 2.9 | A | . |  | . |  | . |  |
| **Bivalvia** | 0.0 | A | 2.1 | A | 1.1 | A | 0.0 | A | 0.0 | A | 0.4 | A |
| Sphaeriidae | 0.0 |  | 2.1 |  | 1.1 |  | 0.0 |  | 0.0 |  | 0.4 |  |
| **Cladocera** | . |  | . |  | . |  | 0.0 | A | Trace | A | 0.0 | A |
| **Coleoptera** | 28.0 | A | 6.2 | B | 7.8 | B | 11.8 | A | 8.8 | A | 1.8 | A |
| Heteroceridae | 18.3 |  | 0.0 |  | 2.4 |  | 11.8 |  | 8.6 |  | 1.8 |  |
| Hydrophilidae | 9.7 |  | 6.2 |  | 5.4 |  | . |  | . |  | . |  |
| Staphylinidae | . |  | . |  | . |  | 0.0 |  | 0.2 |  | 0.0 |  |
| **Diptera** | 63.7 | A | 15.5 | B | 19.4 | B | 82.4 | A | 17.1 | B | 36.3 | B |
| Ceratopogonidae | 20.7 |  | 3.5 |  | 3.1 |  | 14.0 |  | 0.7 |  | 0.6 |  |
| Chironomidae | 27.9 |  | 8.8 |  | 11.6 |  | 62.4 |  | 15.3 |  | 31.0 |  |
| Dolichopodidae | 0.0 |  | 3.1 |  | 4.0 |  | 0.0 |  | 0.3 |  | 1.5 |  |
| Empididae | 7.4 |  | 0.0 |  | 0.0 |  | . |  | . |  | . |  |
| Ephydridae | 7.7 |  | 0.1 |  | 0.8 |  | 0.0 |  | 0.9 |  | 3.2 |  |
| Muscidae | . |  | . |  | . |  | 5.9 |  | 0.0 |  | 0.0 |  |
| **Gastropoda** | 0.0 | A | 2.9 | A | 1.8 | A | 0.0 | A | 0.0 | A | 2.1 | A |
| Lymnaeidae | . |  | . |  | . |  | 0.0 |  | 0.0 |  | 2.1 |  |
| Physidae | 0.0 |  | 2.9 |  | 1.8 |  | . |  | . |  | . |  |
| **Hemiptera** | 0.3 | A | 1.6 | A | 0.9 | A | 0.0 | A | 0.0 | A | 4.0 | A |
| Corixidae | 0.0 |  | 1.6 |  | 0.9 |  | 0.0 |  | 0.0 |  | 4.0 |  |
| **Hirudinea** | 4.5 | A | 3.6 | A | 2.6 | A | 0.0 | A | 0.0 | A | 3.7 | A |
| Glossiphonidae | 4.5 |  | 3.6 |  | 2.6 |  | 0.0 |  | 0.0 |  | 3.7 |  |
| **Isopoda** | 0.0 | A | 1.1 | A | 0.0 | A | 0.0 | A | 0.2 | A | 4.1 | A |
| **Nematoda** | 0.0 | A | 1.0 | B | 0.3 | A | 0.0 | A | 0.6 | A | 6.1 | A |
| **Oligochaeta** | 3.5 | A | 60.2 | B | 59.3 | B | 5.9 | A | 72.7 | B | 41.5 | C |
| **Ostracoda** | 0.3 | A | 5.5 | B | 0.3 | A | 0.0 | A | T | A | 0.0 | A |
| **Trichoptera** | 0.0 | A | 0.4 | A | 3.5 | A | 0.0 | A | 0.5 | A | 0.0 | A |
| Leptoceridae | 0.0 |  | 0.4 |  | 3.5 |  | 0.0 |  | 0.5 |  | 0.0 |  |
