# Supplementary material for: Foraging Ecology of Fall-Migrating Shorebirds in the Illinois River Valley
Source: PLoS One. 2012 Sep 18;7(9):e45121. doi: 10.1371/journal.pone.0045121 (PMC3445572; doi:10.1371/journal.pone.0045121)
Supplement: Table S3 — Aggregate percent mass (dry) of taxa found in fall migrating Lesser Yellowlegs ingesta and core samples taken at collection and random sites in 2007 ( n = 34) and 2008 ( n = 20). Values with different letters within Taxa Orders (rows) indicate significant differences of least-squares means (Tukey-Kramer test: P≤0.05). (DOCX) [file pone.0045121.s003.docx]

Table S3.

|  |  |  |  |  |  |  |  |  |  |  |  |  |
| --- | --- | --- | --- | --- | --- | --- | --- | --- | --- | --- | --- | --- |
|  | 2007 | | | | | | 2008 | | | | | |
| Order | Diet | | Collection | | Random | | Diet | | Collection | | Random | |
| **Bivalvia** | 0.0 | A | 0.6 | A | 0.4 | A | 4.2 | A | 0.0 | A | 2.1 | A |
| Sphaeriidae | 0.0 |  | 0.6 |  | 0.4 |  | 4.2 |  | 0.0 |  | 2.1 |  |
| **Cladocera** | 0.0 | A | T | A | 0.0 | A | 0.0 | A | 0.1 | A | 0.0 | A |
| **Coleoptera** | 23.7 | A | 0.4 | B | 3.2 | B | 0.3 | A | 0.0 | A | 0.0 | A |
| Chrysomelidae | 1.5 |  | 0.0 |  | 0.0 |  | . |  | . |  | . |  |
| Heteroceridae | 5.4 |  | 0.0 |  | 0.0 |  | . |  | . |  | . |  |
| Hydrophilidae | 16.8 |  | 0.4 |  | 3.2 |  | 0.3 |  | 0.0 |  | 0.0 |  |
| **Diptera** | 24.4 | A | 33.5 | A | 21.9 | A | 31.4 | A | 29.6 | A | 34.0 | A |
| Ceratopogonidae | 1.4 |  | 2.5 |  | 1.7 |  | 0.0 |  | 0.2 |  | 0.4 |  |
| Chironomidae | 23.0 |  | 30.2 |  | 18.3 |  | 31.4 |  | 28.3 |  | 33.5 |  |
| Dolichopodidae | 0.0 |  | 0.3 |  | 1.2 |  | . |  | . |  | . |  |
| Sciomyzidae | 0.0 |  | 0.0 |  | 0.6 |  | 0.0 |  | 1.1 |  | 0.0 |  |
| Syrphidae | 0.0 |  | 0.0 |  | 0.1 |  | . |  | . |  | . |  |
| **Ephemeroptera** | 2.7 | A | 0.0 | A | 0.0 | A | . |  | . |  | . |  |
| Baetidae | 2.7 |  | 0.0 |  | 0.0 |  | . |  | . |  | . |  |
| **Fish** | . |  | . |  | . |  | 19.6 | A | 0.0 | B | 0.0 | B |
| *Gambusia spp.* | . |  | . |  | . |  | 19.6 |  | 0.0 |  | 0.0 |  |
| **Gastropoda** | 0.0 | A | 5.7 | A | 5.6 | A | 0.0 | A | 0.0 | A | 1.0 | A |
| Physidae | 0.0 |  | 0.9 |  | 3.2 |  | 0.0 |  | 0.0 |  | 0.7 |  |
| Planorbidae | 0.0 |  | 4.9 |  | 2.4 |  | 0.0 |  | 0.0 |  | 0.2 |  |
| **Hemiptera** | 18.9 | A | 3.4 | B | 3.2 | B | 12.8 | A | 0.1 | A | 4.3 | A |
| Corixidae | 18.9 |  | 3.3 |  | 3.2 |  | 12.8 |  | 0.1 |  | 4.3 |  |
| Mesoveliidae | 0.0 |  | 0.1 |  | 0.0 |  | . |  | . |  | . |  |
| **Hirudinea** | 4.0 | A | 5.4 | A | 9.1 | A | 0.0 | A | 4.4 | A | 3.7 | A |
| Glossiphonidae | 4.0 |  | 5.4 |  | 9.1 |  | 0.0 |  | 4.4 |  | 3.7 |  |
| **Isopoda** | 0.0 | A | 0.0 | A | 0.1 | A | 1.6 | A | 1.4 | A | 1.1 | A |
| **Nematoda** | 3.6 | A | 3.0 | A | 1.1 | A | 22.5 | A | 0.5 | B | 0.4 | B |
| **Oligochaeta** | 0.0 | A | 46.8 | B | 53.4 | B | 5.0 | A | 57.3 | B | 53.3 | B |
| **Ostracoda** | 13.4 | A | 0.2 | B | 0.2 | B | 2.6 | A | 0.0 | A | 0.0 | A |
| **Trichoptera** | 9.5 | A | 1.0 | A | 1.8 | A | 0.0 | A | 6.5 | A | 0.2 | A |
| Leptoceridae | 9.5 |  | 1.0 |  | 1.8 |  | 0.0 |  | 6.5 |  | 0.2 |  |
|  |  |  |  |  |  |  |  |  |  |  |  |  |
