# Supplementary material for: Foraging Ecology of Fall-Migrating Shorebirds in the Illinois River Valley
Source: PLoS One. 2012 Sep 18;7(9):e45121. doi: 10.1371/journal.pone.0045121 (PMC3445572; doi:10.1371/journal.pone.0045121)
Supplement: Table S4 — Aggregate percent mass (dry) of taxa found in Pectoral Sandpiper ingesta and core samples taken at collection and random sites in 2007 ( n = 37) and 2008 ( n = 28). Values with different letters within Taxa Orders (rows) indicate significant differences of least-squares means (Tukey-Kramer test: P≤0.05). (DOCX) [file pone.0045121.s004.docx]

Table S4.

|  |  |  |  |  |  |  |  |  |  |  |  |  |
| --- | --- | --- | --- | --- | --- | --- | --- | --- | --- | --- | --- | --- |
|  | 2007 | | | | | | 2008 | | | | | |
| Taxa | Diet | | Collection | | Random | | Diet | | Collection | | Random | |
| **Arachnida** | 0.0 | A | T | A | 0.0 | A | 0.0 | A | 0.0 | A | 0.1 | A |
| **Bivalvia** | 0.0 | A | 3.0 | A | 2.6 | A | 3.4 | A | 3.6 | A | 0.9 | A |
| Sphaeriidae | 0.0 |  | 3.0 |  | 2.6 |  | 3.4 |  | 3.6 |  | 0.9 |  |
| **Cladocera** | . |  | . |  | . |  | 0.0 | A | T | A | 0.0 | A |
| **Coleoptera** | 3.7 | A | 0.4 | A | 4.2 | A | 0.0 | A | 0.5 | A | 0.0 | A |
| Chrysomelidae | 0.0 |  | 0.4 |  | 0.0 |  | 0.0 |  | 0.5 |  | 0.0 |  |
| Hydrophilidae | 3.7 |  | 0.0 |  | 4.2 |  | . |  | . |  | . |  |
| **Diptera** | 67.1 | A | 38.3 | B | 30.7 | B | 72.9 | A | 24.3 | B | 21.7 | B |
| Ceratopoginidae | 3.2 |  | 4.9 |  | 1.8 |  | 2.1 |  | 0.4 |  | 0.2 |  |
| Chironomidae | 63.9 |  | 32.1 |  | 27.1 |  | 65.5 |  | 21.7 |  | 20.1 |  |
| Dolichopodidae | 0.0 |  | 0.1 |  | 0.8 |  | 0.0 |  | 0.2 |  | 0.0 |  |
| Empididae | 0.0 |  | 0.7 |  | 0.0 |  | 0.9 |  | 0.0 |  | 0.0 |  |
| Ephydridae | 0.0 |  | 0.4 |  | 0.4 |  | 0.0 |  | 0.6 |  | 1.3 |  |
| Muscidae | . |  | . |  | . |  | 4.3 |  | 0.0 |  | 0.0 |  |
| Phoridae | 0.0 |  | 0.1 |  | 0.0 |  | . |  | . |  | . |  |
| Sciomyzidae | 0.0 |  | 0.0 |  | 0.3 |  | 0.0 |  | 0.4 |  | 0.0 |  |
| Stratiomyidae | 0.0 |  | 0.0 |  | 0.3 |  | . |  | . |  | . |  |
| Tabanidae | . |  | . |  | . |  | 0.0 |  | 1.1 |  | 0.0 |  |
| **Gastropoda** | 2.7 | A | 5.5 | A | 2.2 | A | 0.0 | A | 1.5 | A | 0.4 | A |
| Physidae | 2.7 |  | 0.7 |  | 2.2 |  | 0.0 |  | 1.5 |  | 0.4 |  |
| Planorbidae | 0.0 |  | 4.8 |  | 0.0 |  | . |  | . |  | . |  |
| **Hemiptera** | 13.6 | A | 3.8 | B | 0.9 | B | 0.0 | A | 0.1 | AB | 0.7 | B |
| Corixidae | 13.6 |  | 3.8 |  | 0.9 |  | 0.0 |  | 0.1 |  | 0.7 |  |
| **Hirudinea** | 0.0 | A | 3.7 | A | 5.2 | A | 0.0 | A | 0.6 | A | 0.0 | A |
| Glossiphonidae | 0.0 |  | 3.7 |  | 5.2 |  | 0.0 |  | 0.6 |  | 0.0 |  |
| **Isopoda** | 0.0 | A | 0.0 | A | 0.2 | A | 19.8 | A | 2.5 | B | 4.1 | B |
| **Nematoda** | 7.2 | A | 1.7 | A | 2.6 | A | 3.8 | A | 0.5 | A | 0.7 | A |
| **Oligochaeta** | 1.5 | A | 41.1 | B | 50.5 | B | 0.0 | A | 65.9 | B | 71.5 | B |
| **Ostracoda** | 2.7 | A | 0.8 | A | T | A | 0.0 | A | T | A | 0.0 | A |
| **Trichoptera** | 1.5 | A | 1.8 | A | 0.8 | A | 0.0 | A | 0.7 | A | 0.0 | A |
| Leptoceridae | 1.5 |  | 1.8 |  | 0.8 |  | 0.0 |  | 0.7 |  | 0.0 |  |
|  |  |  |  |  |  |  |  |  |  |  |  |  |
